# Two Small Peptides from *Buthus martensii* Hydrolysates Exhibit Antitumor Activity Through Inhibition of TNF-α-Mediated Signal Transduction Pathways

**DOI:** 10.3390/life15010105

**Published:** 2025-01-15

**Authors:** Mengshuang Zhu, Shanshan Zhang, Jiyang Tang, Hairong Hou, Lizhen Wang, Houwen Lin, Xuanming Zhang, Meng Jin

**Affiliations:** 1 Engineering Research Center of Zebrafish Models for Human Diseases and Drug Screening, Biology Institute, Qilu University of Technology (Shandong Academy of Sciences), Jinan 250103, China; zms1967489150@163.com (M.Z.); qingshuibaikai@126.com (S.Z.); tangjiyang0331@163.com (J.T.); caomu_1314@163.com (H.H.); wlzh1106@126.com (L.W.); franklin67@126.com (H.L.); 2 Research Center for Marine Drugs, State Key Laboratory of Oncogenes and Related Genes, Department of Pharmacy, School of Medicine, Shanghai Jiao Tong University, Shanghai 200127, China

**Keywords:** *Buthus martensii*, small peptides, gastric cancer, TNF/TNFR, molecular mechanism

## Abstract

The scorpion *Buthus martensii* Karsch is edible and has been an essential resource in traditional Chinese medicine for treating numerous diseases. In this study, two small peptides from *B. martensii* hydrolysates were examined to elucidate their potential against gastric cancer. The small peptides (AK and GK) were identified using the LC-QTOF-MS-based approach. In silico prediction of therapeutic targets, MGC-803 cells and transgenic zebrafish models, and immunoblotting experiments were used to reveal the molecular mechanism of action of the peptides. The peptides AK and GK competitively bound to the receptor to modulate the TNF/TNFR-signaling cascade and alter the tumor microenvironment. EGFR, TP53, MYC, PTEN, and STAT3 were also identified as major functional targets of the peptides. Mechanistically, AK and GK inactivated the TNF-α/EGFR/STAT3-signaling pathway, decreased c-myc protein expression levels, and upregulated p53 and PTEN expression, thereby preventing TNF-α-induced tumor growth. Our findings indicated that AK and GK played a pivotal role in offsetting the inflammatory stimuli that caused gastric cancer cell invasion and highlighted the use of *B. martensii* resources as functional products with health benefits.

## 1. Introduction

Gastric carcinoma arises from the intricate interplay between microbial, environ-mental, and host genetic factors and is one of the leading causes of cancer-related death worldwide [1,2]. The highest incidence rates of gastric carcinoma have been reported in East Asia, which encompasses China, Korea, and Japan, with a yearly incidence rate ranging between 40 and 60 per 100,000 population. For the treatment of gastric cancer, the tumor is usually removed surgically as the primary intervention, followed by chemotherapy to minimize the risk of future tumor formation. However, artificial chemotherapy drugs often come with significant toxic side effects in clinical trials. Natural chemical substances from plants, animals, and microorganisms have been investigated for their potential benefits in treating diseases, as they generally exhibit lower toxicity levels and provide long-term advantages. Natural products offer a plethora of novel prototypes with pharmacological activity, which are believed to possess various anticancer properties that affect cellular apoptosis, differentiation, and angiogenesis [3,4]. In addition, the updated studies have increasingly highlighted the role of persistent and unresolved inflammation in the progression of gastric carcinoma as well as other cancers. A central molecule in the inflammatory microenvironment is the cytokine tumor necrosis factor-alpha (TNF-α). Although high-dose TNF-α has been utilized as a cytotoxic agent over the past few decades, recent preclinical cancer models support the link between chronic, low-level exposure to TNF-α, and the development of pro-malignant phenotypes [5,6]. Overall, therapeutic approaches employing natural agents to modulate the microenvironment are presently under evaluation in medical studies.

The scorpion *Buthus martensii* (*B. martensii*) Karsch, a widely distributed species in China, is edible and has been an essential resource in traditional Chinese medicine for thousands of years. Whole scorpions and scorpion tails have been shown to be effective in the treatment of many diseases, such as tumors, hepatopathy, neurological diseases, and cardiovascular diseases [7,8,9]. Bioactive peptides with 13–76 amino acid residues are the most important components of scorpion venom in the tail. These peptides can be separated into two main types: disulfide-bridged peptides (DBPs) and non-disulfide-bridged peptides (NDBPs). DBPs generally interact with membrane-bound ion channels (Na^+^, K^+^, Ca^2+^, and Cl^−^ channels), which are essential for regulating normal cellular physiology in various mammalian organisms. Unlike the DBPs that display conserved structure–function relationships, the NDBPs are structurally diverse and demonstrate activity against a wide range of biological targets [10,11]. The scorpion body is also considered to be a rich source of biologically active peptides. In 2014, Ren et al. [12] identified a novel anticoagulant peptide from scorpion protein hydrolysates. However, until now, systematic information regarding whole scorpion peptides has been limited. In the present study, two small peptides were identified from *B. martensii* hydrolysates via liquid chromatography–quadrupole time-of-flight–mass spectrometry (LC-QTOF-MS). In silico prediction of therapeutic targets, in vitro and in vivo biological assessment, and immunoblotting experiments were used to determine the role of these peptides in the treatment of gastric carcinoma. Our results highlighted the use of *B. martensii* peptides as functional products with health benefits.

## 2. Materials and Methods

### 2.1. Chemicals and Materials

Dry scorpions (*B. martensii*, 5–6 cm in length) were obtained from Linyi (Shandong Province, China). Samples were selected by Professor Kechun Liu at the Biology Institute, and a sample specimen (No. Q2021137) was deposited at the Key Laboratory for Drug Screening Technology (Biology Institute, Qilu University of Technology, Shandong Academy of Sciences). Trypsin (1:250) was purchased from Beijing Solarbio Science & Technology Co., Ltd. (Beijing, China). Ultrapure grade water and acetonitrile were obtained from Watsons Ltd. (Hong Kong, China) and Tedia Company Inc. (Fairfield, OH, USA), respectively. The small peptides Ala-Lys (AK) and Gly-Lys (GK) were purchased from Shanghai Dechi Biosciences Co., Ltd. (Shanghai, China). Tumor necrosis factor (TNF)-α and CuSO_4_ were obtained from Abcam Biotechnology (Cambridge, UK) and Sinopharm Chemical Reagent Co., Ltd. (Beijing, China), respectively. Astragalus polysaccharides were purchased from Shanghai Yuanye Biotechnology Co., Ltd. (Shanghai, China).

### 2.2. Preparation of B. martensii Hydrolysates

Enzymatic hydrolysis of *B. martensii* was performed by incubating 1 g of the sample with 8 mL water and 1200 U/g trypsin for 1 h at 50 °C. The hydrolyzed solutions were heated for a further 15 min at 85 °C, then fractionated using an ultrafiltration membrane with 10 kDa molecular weight cut-off. The filtrate was concentrated and freeze-dried, and 125.1 mg of hydrolysate powder was obtained.

### 2.3. LC-MS-Based Identification of Small Peptides

*B. martensii* hydrolysates (3 mg/mL) were analyzed using an Agilent 1260 HPLC-6530 QTOF analyzer equipped with an Agilent Eclipse XDB-C_18_ column (4.6 × 250 mm, 5 μm). LC-MS/MS analysis was performed under the following conditions: mobile phase water (A) and acetonitrile (B) at 1 mL/min: 2% B for 0–5 min, 2–15% B for 5–25 min, 15–30% B for 25–40 min, 30–35% B for 40–50 min, and 35–60% B for 50–55 min. The optimized MS settings were 100–2000 *m*/*z* in positive ionization modes, nebulizer pressure 40 psi, gas temperature 350 °C, drying gas flow rate 12 L/min, and capillary voltage 3500 V. The Mascot search engine from Matrix Science (http://www.matrixscience.com, (accessed on 26 August 2023)) was used to search the acquired MS data and identify peptides. MS fragmentation and chromatographic retention were manually examined for qualitative small peptide analysis.

### 2.4. In Silico Prediction of Therapeutic Targets

The chemical structures of AK and GK were downloaded from the PubChem da-tabase (https://pubchem.ncbi.nlm.nih.gov/ (accessed on 17 March 2024)). Swiss Target Prediction (http://www.swisstargetprediction.ch/ (accessed on 17 March 2024)) was used to predict the targets associated with these peptides. KEGG pathway enrichment analyses were carried out using OmicShare Tools (https://www.omicshare.com/tools (accessed on 19 March 2024)) [13]. Based on the enrichment scores of the small peptides, “gastric cancer”-related targets were screened using DisGeNET (http://www.disgenet.org/ (accessed on 21 March 2024)) and GeneCards (https://www.genecards.org/ (accessed on 21 March 2024)) databases. After removing redundant entries, candidate targets were analyzed using the String 12.0 database (https://cn.string-db.org/ (accessed on 21 March 2024)) to identify potential protein interactions. Potential target proteins were integrated with Cytoscape to form a peptide–protein interaction (PPI) network. Molecular docking studies were conducted using AutoDock Tools to confirm the interactions between TNF receptor (TNFR) and the peptides. The crystal structure of human TNFR (PDB ID: 1TNR) was downloaded from the Protein Data Bank (https://www.rcsb.org/ (accessed on 28 April 2024)). The molecular docking analysis was carried out by removing non-standard residues, adding hydrogen, and calculating Gasteiger charges under the default parameters [14]. The molecular affinities of ligand–macromolecule were analyzed and visualized using the PyMol 2.5 package.

### 2.5. Assessment of the Inflammatory Microenvironment In Vitro and In Vivo

MGC-803 cells (American Type Culture Collection, Manassas, VA, USA) were cultured in DMEM containing 10% fetal bovine serum. Cells (5 × 10^3^ cells per well) were seeded into 96-well plates, and the antiproliferative effects of AK and GK were evaluated using an MTT assay [15]. Cells were divided into different treatment groups: control, model (TNF-α 0.5 ng/mL), positive (TNF-α 0.5 ng/mL + APS 20 μg/mL), and intervention (TNF-α 0.5 ng/mL + AK or GK at 0.5~100 μM concentrations). Cell viability was assessed using a Multiskan FC microplate reader (Thermo Scientific, Waltham, MA, USA) to measure the absorbance at 595 nm.

The in vivo activities of AK and GK were assessed in Tg (zlyz:EGFP) transgenic zebrafish as previously described [16]. Zebrafish larvae (3 dpf) were placed in 6-well plates and treated with AK or GK at concentrations of 10, 25, and 50 μM, followed by incubation with 20 μM CuSO_4_. The effects of the small peptides on inflammation were assessed using a fluorescence microscope (SZX16, Olympus, Tokyo, Japan) to count the number of inflammatory cells migrating towards the zebrafish lateral line.

### 2.6. Assessment of Protein Expression Levels

Protein lysates were extracted from MGC-803 cells and subjected to Western blot analysis as described previously, with minor modifications [17]. Briefly, total proteins were resolved by SDS-PAGE, transferred to PVDF membranes, and probed with the appropriate primary and secondary antibodies. Protein bands were visualized using the Tanon 5200 chemiluminescent imaging system (Tanon Science & Technology, Shanghai, China). The following antibodies were used: anti-EGFR (1:1500, cat No. ab52894, Abcam), anti-p-EGFR (1:1500, cat No. ab32430, Abcam), anti-STAT3 (1:1500, cat No. 10253-2-AP; Proteintech, Rosemont, IL, USA), anti-p-STAT3 (1:1500, cat No. ab76315, Abcam), anti-c-myc (1:1500, cat No. 67447-1-Ig, Proteintech), anti-p53 (1:1500, cat No. 60283-2-Ig, Proteintech), anti-PTEN (1:1500, cat No. 22034-1-AP, Proteintech), anti-β-actin (1:5000, cat No. 20536-1-AP, Proteintech), HRP-conjugated goat anti-rabbit IgG (1:5000, cat No. A0208, Beyotime, China), and HRP-conjugated goat anti-mouse IgG (1:5000, cat No. A0216, Beyotime).

### 2.7. Statistical Analysis

In biological assays for multiple comparisons, ANOVA tests were performed using an online platform, OmicShare Tools (https://www.omicshare.com/tools (accessed on 8 June 2024)). All data were uploaded and analyzed using Fisher’s least significant difference (LSD) method. A *p*-value of < 0.05 was considered to be statistically significant.

## 3. Results and Discussion

### 3.1. Identification of Small Peptides in B. martensii Hydrolysates

**Figure 1:**
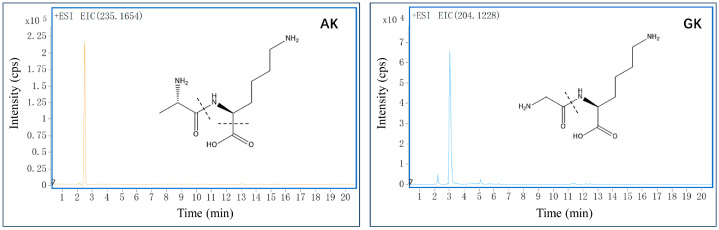
Identification of the small peptides AK and GK using the Mascot search engine and MS/MS analyses.

Using the LC-MS-based approach, we captured two small peptides from *B. martensii* hydrolysates (Figure 1). The peaks *m*/*z* 235.1654 ([M + NH_4_]^+^, 2.72 min) and *m*/*z* 204.1228 ([M + H]^+^, 3.03 min) were retrieved from the SwissProt database using the Mascot search engine and identified as Ala-Lys (AK) and Gly-Lys (GK), respectively. Subsequent MS/MS analyses revealed that AK produced the main fragment ion at *m*/*z* 118 (Lys—COOH), while GK generated the high intensity fragment ion at *m*/*z* 145 (Lys). Our findings were further confirmed by comparing their retention times with those of the reference substances in the LC-MS analysis (Figures S1 and S2).

### 3.2. Bioinformatics Target Analysis

**Figure 2:**
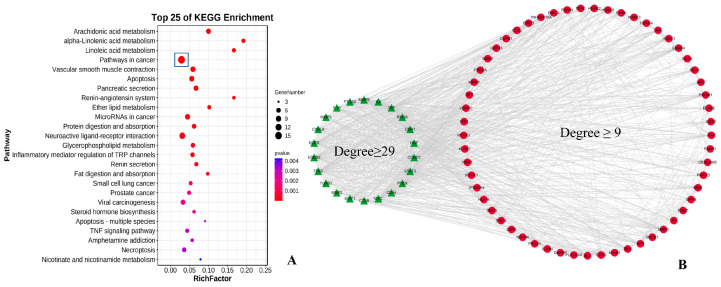
KEGG analysis (**A**) and core target prediction (**B**) for AK and GK. Targets were classified based on the “Degree” score: the green triangle nodes (Degree ≥ 29) and the red circular nodes (9 ≤ Degree < 29).

Following the removal of redundant genes, we next used the Swiss Target Predic-tion database to identify 90 potential targets of the small peptides (AK and GK). KEGG enrichment analysis was conducted to examine the primary functions of the identified targets. Based on the gene number and *p*-value, “pathways in cancer” was found to be the top significant pathway (Figure 2A).

Historically, *B. martensii* is an important resource in Chinese traditional medicine and has been used to treat gastrointestinal tumors [18]. Here, we obtained a total of 92 critical genes associated with gastric cancer using DisGeNET and GeneCards databases. Following integration of the peptide and disease targets, the String database was used to identify protein–protein interactions in order to determine the association between the peptides and disease. As shown in Figure 2B, the visualized network system was mapped with 79 nodes and 879 edges to examine the underlying processes of AK and GK in the treatment of gastric cancer. Degree scores were determined based on the number of edges involved, which were used to classify the importance of the targets. An in silico topological analysis revealed that there were 22 genes with degree scores of ≥29 (the green triangle) and 57 genes with degree scores of ≥9 and <29 (the red circular). TNF (Degree, 72) was the principal target of peptide-mediated antitumor therapy. TNF-α is a member of the TNF/TNFR superfamily, and previous clinical trials have shown that it is a proinflammatory cytokine associated with a range of tumor types [19,20]. EGFR (Degree, 66), TP53 (Degree, 65), MYC (Degree, 58), PTEN (Degree, 52), and STAT3 (Degree, 50) were also identified as major functional targets of AK and GK (Table S1). Subsequent molecular docking analysis, bioactive assays, and immunoblotting experiments confirmed the role of AK and GK in modifying the microenvironment and intracellular interactions via these signaling targets, further elucidating the mechanism of action of these peptides in gastric cancer.

### 3.3. Binding of the Peptides to TNFR

**Figure 3:**
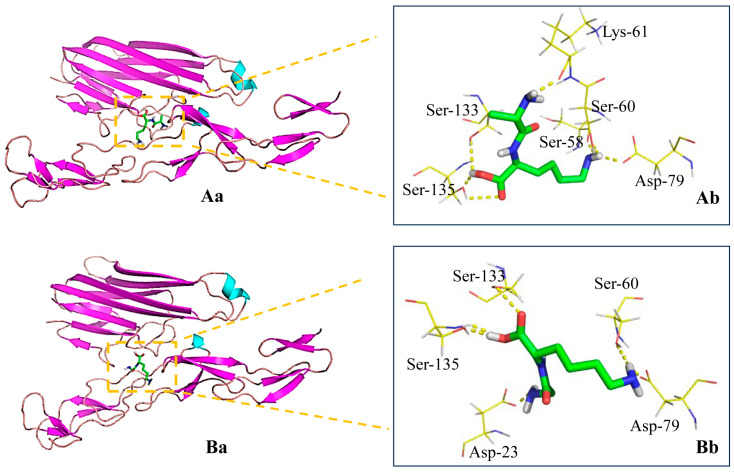
Molecular docking analysis of AK (**A**) and GK (**B**). Docked poses (**a**) and H-bonding interactions (**b**) between the peptides and macromolecules.

Molecular docking analysis was used to simulate the docking of AK and GK to TNFR to identify the specific function of the molecules. The binding poses, H-bonding interactions, and docking scores were visualized and can be found in Figure 3 and Table S2. AK and GK were found to interact strongly with the TNFR active pocket and exhibited binding-free energies of −3.04 kcal/mol and −3.13 kcal/mol, respectively. AK was found to form seven hydrogen bond interactions with six residues (one with Ser-58, one with Ser-60, one with Lys-61, one with Asp-79, one with Ser-133, and two with Ser-135), while GK yielded six hydrogen bond interactions with five residues (one with Asp-23, one with Ser-60, one with Asp-79, one with Ser-133, and two with Ser-135). These docking simulations demonstrated that the two peptides presented high potential affinities with TNFR and competitively bound to the receptor. TNF-α antagonists have previously been shown to be effective candidates for modulating the inflammatory microenvironment, thereby revolutionizing the treatment of cancer [21].

### 3.4. Effects of AK and GK on the Inflammatory Microenvironment

TNF-α is a proinflammatory cytokine involved in regulating tumor progression. Here, we examined the effects of AK and GK against TNF-α-induced proliferation in MGC-803 human gastric cancer cells (Figure S3). Cell viability was significantly increased following treatment with 0.5 ng/mL TNF-α, highlighting its potent pro-tumorigenic properties. However, tumor cell growth was inhibited in the AK and GK treatment groups (0.5~100 μM for 48 h), with statistically significant differences observed at concentrations of 2.5~100 μM (*p* < 0.05) compared with the model group. These findings suggested that treatment with AK and GK reversed TNF-α-induced proliferation of MGC-803 cells, which is critical for gastric cancer therapy.

**Figure 4:**
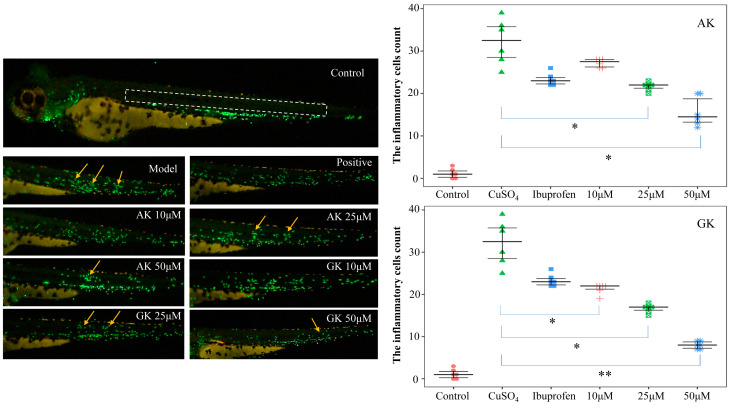
Effects of AK and GK on the inflammatory microenvironment. (* *p* < 0.05, ** *p* < 0.01 versus the model group). The white dotted box represented the area where inflammatory cells were observed. The yellow arrows indicated the aggregation of inflammatory cells.

Next, the effects of AK and GK (10, 25 and 50 μM) on inflammatory cell recruitment were examined in vivo using transgenic zebrafish embryos (Figure 4). Incubation with CuSO_4_ led to significant migration of inflammatory cells to the site of the zebrafish lateral line, indicating that the inflammation model had been successfully established. Treatment with AK and GK resulted in inhibition of inflammatory cell migration with statistically significant differences observed at 25~50 μM (AK) and 10~50 μM (GK). The effects of 50 μM GK were particularly superior to that of the positive control drug. Inflammation serves as a key driver in the formation of the tumor microenvironment. The persistent inflammatory state is crucial for the progression from chronic atrophic gastritis to gastric cancer. Additionally, inflammatory cells along with their products, such as cytokines, may facilitate tumor development during the initiation, progression, and metastasis phases [22,23]. Our findings confirmed that the small peptides AK and GK have the potential to reduce the inflammatory microenvironment.

### 3.5. The Effects of AK and GK on Protein Expression Levels in MGC-803 Cells

**Figure 5:**
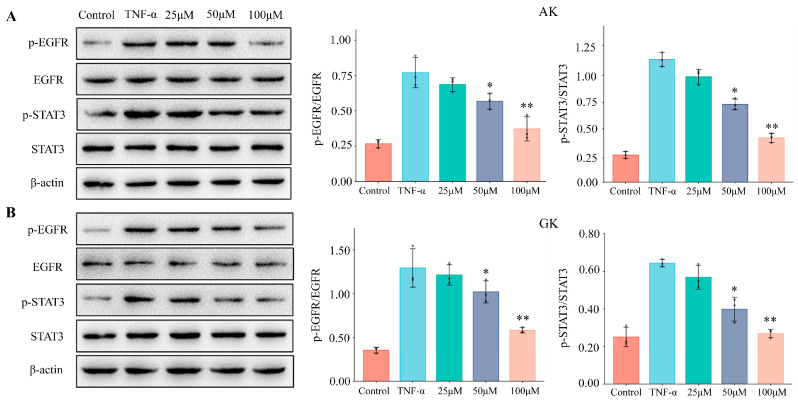
Effects of AK (**A**) and GK (**B**) on total and phosphorylated EGFR and STAT3 protein expression levels. (* *p* < 0.05, ** *p* < 0.01 versus the TNF-α group).

Epidermal growth factor receptor (EGFR) is a member of the receptor tyrosine ki-nase family and a downstream-signaling target of TNF-α [24]. EGFR has been shown to activate the phosphatidylinositol 3-kinase (PI3K) and MAPK pathways to regulate cell proliferation, migration, and angiogenesis, which are closely associated with tumor growth and invasion [25]. The signal transducer and activator of transcription (STAT) protein family modulates gene transcription and comprises seven members. STAT3 is an important mediator for the initiation of inflammation and cellular transformation in numerous tumors. STAT3 activation may promote gastric tumorigenesis by both preventing apoptosis and enhancing cell proliferation [26,27]. Here, we found an increase in p-EGFR and p-STAT3 protein expression levels following TNF-α treatment (Figure 5), while p-EGFR/EGFR and p-STAT3/STAT3 ratios were also significantly increased, demonstrating that TNF-α treatment leads to activation of EGFR and STAT3 in MGC-803 cells. In the intervention groups, treatment with AK and GK blocked the phosphorylation of EGFR and STAT3. Our Western blotting data revealed a dose-dependent decrease in protein expression levels at concentrations of 25–100 μM. Moreover, previous studies have indicated that EGFR may promote tumor growth via STAT3 activation [28,29]. Our data confirmed a functional role for the TNF-α/EGFR/STAT3-signaling pathway in the proliferation of tumor cells, as well as demonstrated that AK and GK could block TNF-α-induced activation of EGFR and STAT3 by acting as potent antagonists. Thus, AK and GK may have therapeutic potential in the prevention of gastric cancer.

**Figure 6:**
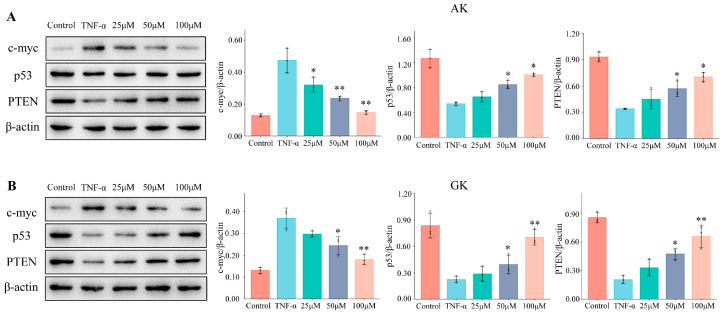
Effects of AK (**A**) and GK (**B**) on c-myc, p53, and PTEN protein expression levels. (* *p* < 0.05, ** *p* < 0.01 versus the TNF-α group).

Cellular myelocytomatosis oncogene (c-myc) is frequently aberrantly amplified in various human tumors, leading to the occurrence and development of cancers. c-Myc can also be induced by TNF-α and has been recognized as a critical regulator of the tumor microenvironment [30]. Here, TNF-α-induced proliferation of MGC-803 human gastric cancer cells led to increased c-myc expression levels, which was associated with enhanced tumor cell growth (Figure 6). Treatment with AK and GK led to decreased c-myc protein expression levels and a corresponding reduction in tumor cell growth. Tumor protein 53 (p53) can activate or repress a range of effector genes associated with anti-proliferative activities, which contribute to its tumor-suppressive properties. The tumor suppressor function of p53 may be influenced by the microenvironment during the course of tumor development [31]. Phosphatase and tensin homolog (PTEN) has also been identified as a cell growth suppressor, and its expression is often lost in tumors [32]. Here, we found that p53 and PTEN were significantly downregulated in MGC-803 cells treated with TNF-α, while treatment with AK and GK led to a dose-dependent increase in p53 and PTEN expression levels at concentrations of 25–100 μM. According to previous reports, TNF-α-induced activation of NF-κB suppresses PTEN expression, and a negative correlation between the activation of the NF-κB and p53 pathways has also been emphasized [33,34]. Our results supported that the TNF-α/NF-κB pathway might be mediated by small peptides AK and GK as antagonists. The findings indicated that the effects of AK and GK were pivotal in reducing the inflammatory stimuli that cause gastric cancer cell invasion.

## 4. Conclusions

*B. martensii* has been recognized as both an edible species and a valuable resource for therapeutic applications. In the present study, two small peptides (AK and GK) were obtained from *B. martensii* through hydrolysis with gastrointestinal enzymes and identified using an LC-MS-based approach. KEGG enrichment analyses indicated that the peptides were predominantly associated with cancer-related pathways. TNF/TNFR were identified as primary bioactive targets that may mediate the inhibitory effects of AK and GK on gastric cancer. TNF-α is a well-established critical regulator of inflammatory signaling cascades. Here, AK and GK were found to display high potential affinities with TNFR and acted as TNF-α antagonists. The potential therapeutic effects of AK and GK were identified using in silico prediction tools. Mechanistically, AK and GK have the potential to reduce the inflammatory response. Specifically, AK and GK inactivated the TNF-α/EGFR/STAT3 signaling pathway, as well as altered the expression of key proteins, including c-myc, p53, and PTEN. Thus, AK and GK may be promising candidates for antitumor therapies. Overall, the current study revealed the modulating effects of *B. martensii* peptides on the tumor microenvironment. Our findings provided a basis for utilizing *B. martensii* as a functional food.

## Data Availability

Data are included within the article and Supplementary Material.

## Supplementary Materials

The following supporting information can be downloaded at https://www.mdpi.com/article/10.3390/life15010105/s1, Figure S1: MS and MS/MS spectra of AK in positive modes; Figure S2: MS and MS/MS spectra of GK in positive modes; Figure S3: Effects of AK and GK against TNF-α induced proliferation of MGC-803 cells. (* *p* < 0.05 versus the TNF-α group); Table S1: Topological parameters for the key targets; Table S2: Binding energy calculation results for TNF receptor.
