# Supplementary material for: Two Small Peptides from Buthus martensii Hydrolysates Exhibit Antitumor Activity Through Inhibition of TNF-α-Mediated Signal Transduction Pathways
Source: Life (Basel). 2025 Jan 15;15(1):105. doi: 10.3390/life15010105 (PMC11766664; doi:10.3390/life15010105)
Supplement: Supplementary file 1 [file life-15-00105-s001.zip › life-3370973-supplementary.pdf]

Supporting information

Two small peptides from *Buthus martensii* hydrolysates exhibit antitumor activity through inhibition of TNF-α-mediated signal transduction pathways

Mengshuang Zhu[1], Shanshan Zhang[1], Jiyang Tang[1], Hairong Hou[1], Lizhen Wang[1], Houwen Lin[1,2], Xuanming Zhang[1,*], Meng Jin[1,*]

[1] *Engineering Research Center of Zebrafish Models for Human Diseases and Drug Screening, Biology Institute, Qilu University of Technology (Shandong Academy of Sciences), Jinan 250103, China*

[2] *Research Center for Marine Drugs, State Key Laboratory of Oncogenes and Related Genes, Department of Pharmacy, School of Medicine, Shanghai Jiao Tong University, Shanghai 200127, China*

*Corresponding author: Xuanming Zhang, zhangmx@sdas.org

Meng Jin, mjin1985@hotmail.com

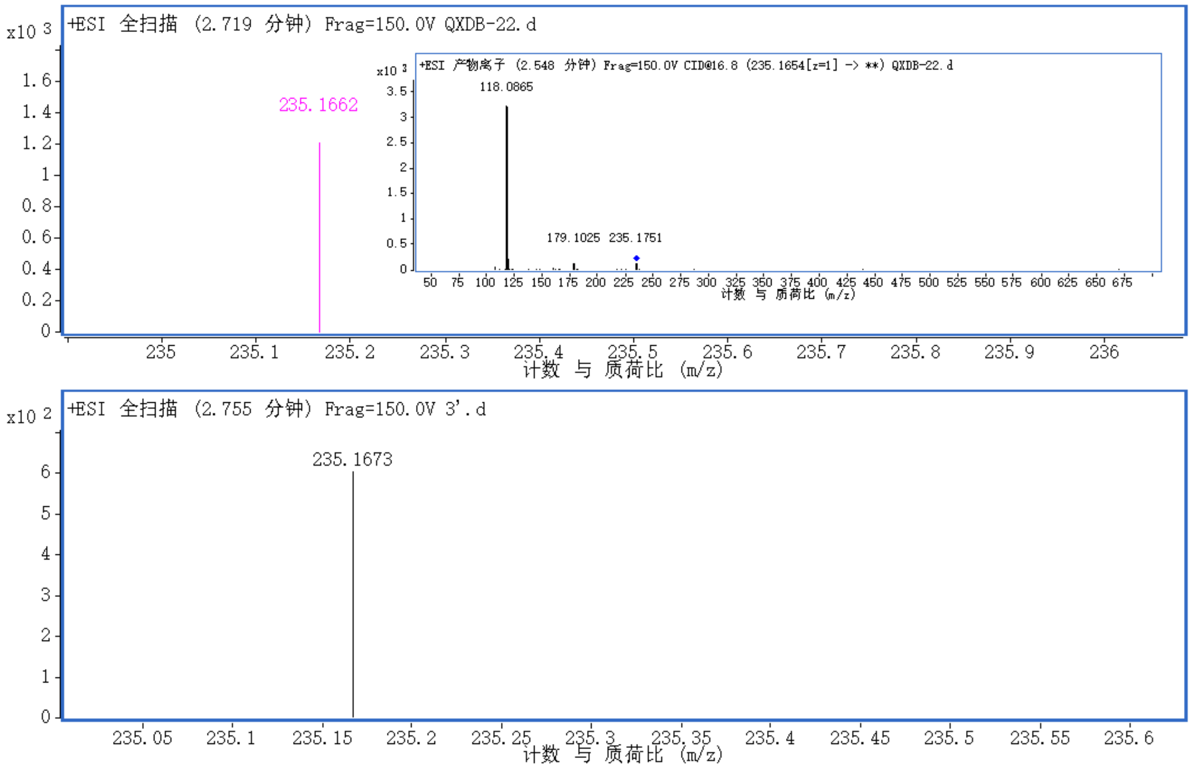

Figure S1 MS and MS/MS spectra of AK in positive modes.

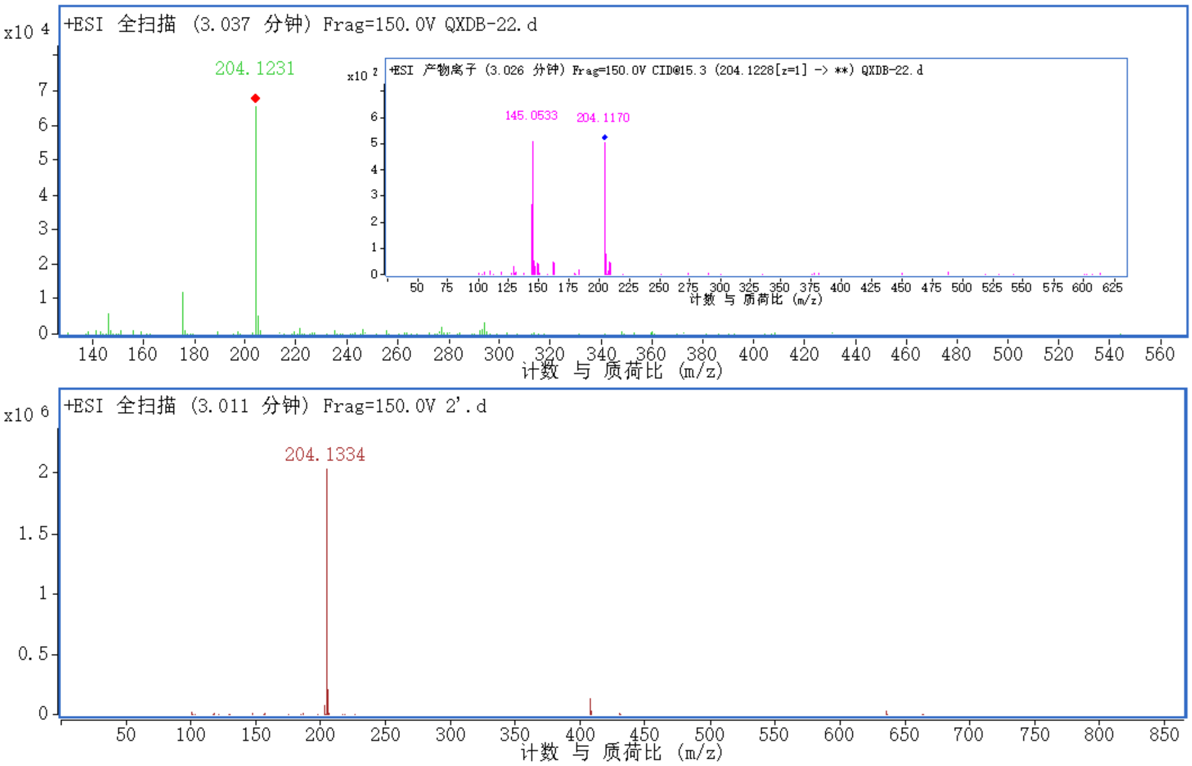

Figure S2 MS and MS/MS spectra of GK in positive modes.

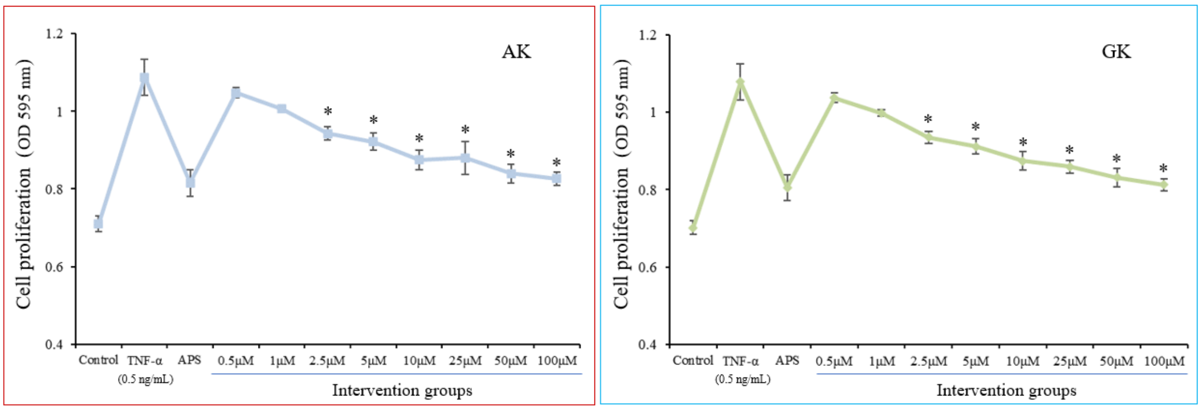

Figure S3 Effects of AK and GK against TNF-α induced proliferation of MGC-803 cells. (*p<0.05 versus the TNF-α group)

Table S1. Topological parameters for the key targets

**Description**	**Gene code**	**Degree**	**Betweenness**	**Closeness**
target	TNF	72	0.086	0.604
target	EGFR	66	0.101	0.594
target	TP53	65	0.084	0.592
target	MYC	58	0.112	0.568
target	PTEN	52	0.044	0.545
target	STAT3	50	0.017	0.539

Table S2. Binding energy calculation results for TNF receptor

Compound	Binding energy ΔG_b (kcal/mol)	Intermolecular energy (kcal/mol)	Electrostatic energy (kcal/mol)	Unbound energy (kcal/mol)
AK	−3.04	−6.03	−1.79	−2.45
GK	−3.13	−6.11	−2.54	−1.31
